# The Impact of COVID-19 Confinement on Cognition and Mental Health and Technology Use Among Socially Vulnerable Older People: Retrospective Cohort Study

**DOI:** 10.2196/30598

**Published:** 2022-02-22

**Authors:** Elena Dura-Perez, Jessica Marian Goodman-Casanova, Amanda Vega-Nuñez, Gloria Guerrero-Pertiñez, Esperanza Varela-Moreno, Maite Garolera, Maria Quintana, Antonio I Cuesta-Vargas, Pilar Barnestein-Fonseca, Carlos Gómez Sánchez-Lafuente, Fermin Mayoral-Cleries, Jose Guzman-Parra

**Affiliations:** 1 Department of Mental Health Regional University Hospital of Málaga Biomedical Research Institute of Malaga (IBIMA) Málaga Spain; 2 Faculty of Psychology University of Málaga Málaga Spain; 3 Brain, Cognition and Behavior: Clinical Research Consorci Sanitari de Terrassa Terrasa Spain; 4 Department of Physiotherapy University of Málaga Biomedical Research Institute of Malaga Málaga Spain

**Keywords:** COVID-19, cognition, quality of life, social isolation, mental health, social support, technology, physical distancing, leisure activities, nursing

## Abstract

**Background:**

COVID-19 forced the implementation of restrictive measures in Spain, such as lockdown, home confinement, social distancing, and isolation. It is necessary to study whether limited access to basic services and decreased family and social support could have deleterious effects on cognition, quality of life, and mental health in vulnerable older people.

**Objective:**

This study aims to explore the impact of the COVID-19 outbreak on cognition in older adults with mild cognitive impairment or dementia as the main outcome and the quality of life, perceived health status, and depression as secondary outcomes and to analyze the association of living alone and a change in living arrangements with those outcomes and other variables related with the use of technology and health services. Likewise, this study aims to analyze the association of high and low technophilia with those variables, to explore the access and use of health care and social support services, and, finally, to explore the informative-, cognitive-, entertainment-, and socialization-related uses of information and communications technologies (ICTs) during the COVID-19 outbreak.

**Methods:**

This cohort study was conducted in Málaga (Spain). In total, 151 participants with mild cognitive impairment or mild dementia, from the SMART4MD (n=75, 49.7%) and TV-AssistDem (n=76, 50.3%) randomized clinical trials, were interviewed by telephone between May 11 and June 26, 2020. All participants had undergone 1-3 assessments (in 6-month intervals) on cognition, quality of life, and mood prior to the COVID-19 breakout.

**Results:**

The outbreak did not significantly impact the cognition, quality of life, and mood of our study population when making comparisons with baseline assessments prior to the outbreak. Perceived stress was reported as moderate during the outbreak. After correction for multiple comparisons, living alone, a change in living arrangements, and technophilia were not associated with negative mental health outcomes. However, being alone was nominally associated with self-perceived fear and depression, and higher technophilia with better quality of life, less boredom, perceived stress and depression, and also less calmness. Overall, health care and social support service access and utilization were high. The most used ICTs during the COVID-19 outbreak were the television for informative, cognitive, and entertainment-related uses and the smartphone for socialization.

**Conclusions:**

Our findings show that the first months of the outbreak did not significantly impact the cognition, quality of life, perceived health status, and depression of our study population when making comparisons with baseline assessments prior to the outbreak. Living alone and low technophilia require further research to establish whether they are risk factors of mental health problems during lockdowns in vulnerable populations. Moreover, although ICTs have proven to be useful for informative-, cognitive-, entertainment-, and socialization-related uses during the pandemic, more evidence is needed to support these interventions.

**Trial Registration:**

ClinicalTrials.gov NCT04385797; https://clinicaltrials.gov/ct2/show/NCT04385797

**International Registered Report Identifier (IRRID):**

RR2-10.2196/26431

## Introduction

COVID-19 was declared a worldwide pandemic by the World Health Organization on March 11, 2020 [1]. To avoid the serious collapse of health systems in response to the rising number of cases and deaths, European countries, as in other continents, decided to implement different measures to control the pandemic.

In Spain, the government decided to declare a national state of alarm, implementing restrictive measures from March 15 until June 21, 2020. The measures included lockdown, home confinement, social distancing, and isolation (activities were limited to basic needs, such as buying food or medication, attending health care centers and financial institutions); closure of schools and nonessential activities; ban of all internal travels except for essential ones; and border closure [2]. These measures also led to a change in health care access: Only critical attention was guaranteed, patient care changed from on-site interviews to telephonic attention, visits with medical specialists were suspended, and there was a lack of monitoring of chronic pathologies.

The elderly population is 1 of the groups most socially vulnerable to this disease. Age alone is by far the most significant factor for death due to COVID-19 [3]. Although COVID-19 infects people of all ages, the risk of becoming seriously ill increases in adults aged over 40 years, and especially in those aged over 60 years. In Spain, 68% of all hospitalizations due to coronavirus and 95% of all deaths correspond to the population over the age of 60 years, with a notable increase after the age of 80 years [4].

Recent data suggest that in addition to old age and medical comorbidities (eg, hypertension, diabetes, obesity), dementia is associated with an increased risk of having severe COVID‐19 and related mortality [5-8]. In addition, restrictive measures, such as confinement, may pose a risk for people with mild cognitive impairment (MCI) or mild dementia (MD). COVID-19 confinement has resulted in an increase in known risk factors for dementia, such as inactivity [9], limited access to basic services [10], isolation [11], and decreased family and social support [12]. These factors could have deleterious effects on cognition, quality of life, and overall health [13]. Therefore, the pandemic has not only a health impact on people with MCI/MD but also a social impact.

Loneliness and social isolation often coexist and are all too common in older adults. Loneliness refers to the subjective state of feeling alone, separated, or apart from others. Social isolation, in contrast, is defined as the objective physical separation from other people, such as living alone, in which one has few social relationships or there is a low frequency of interaction with others [14].

Considering the latter definition, we can understand that the COVID-19 pandemic has increased the social isolation of older adults as restrictive measures have enforced staying at home, distancing, and shutting down all nonessential activities. This has meant that people have been forced to minimize their social interactions to avoid the spread of the virus, leaving those who live alone completely isolated. Social isolation has been identified as a health risk factor as it reduces well-being and is associated with higher prevalence of depression [15] and cognitive impairment [16]. In older adults, it has a greater impact due to decreased social resources, functional and mobility limitations, death of family members, and changes in family structures [17].

During quarantine, factors such as boredom and a lack of activities play an important role. They can contribute to depression [18] and have an impact on the quality of life and functional dependence [19]. Mental activity, in contrast, may improve cognitive function and reduce overall dementia risk [20].

In the “information age,” information and communications technologies (ICTs) have emerged for combating loneliness and social isolation [21]. Although the age-related digital divide and health-related conditions (cognitive, visual, motor, etc) may compromise the use of technologies in the elderly, the extensive home penetration of ICTs has facilitated remote, home-based interventions. These interventions reduce the risk of viral exposure and prevent health-related negative outcomes of social isolation through health care delivery, cognitive stimulation, social connection, information sharing, and leisure entertainment [22].

The aims of this study were (1) to explore the impact of the COVID-19 outbreak on cognition in community-dwelling older adults with MCI/MD as the main outcome and the quality of life, perceived health status, and depression as secondary outcomes; (2) to analyze the differences between individuals living alone and living with others regarding mental health, and other variables related with the use of technology and health services during the COVID-19 outbreak and, likewise, to explore the effect of a change in living arrangements on cognition, quality of life, perceived health status, and depression; (3) to analyze the differences between individuals with high and low technophilia regarding mental health and other variables related with use of technology and health services during the COVID-19 outbreak; (4) to explore the access and use of health care and social support services during the COVID-19 outbreak; and finally (5) to explore the informative-, cognitive-, entertainment-, and socialization-related uses of ICTs during the COVID-19 outbreak.

## Methods

### Study Design

This cohort study was conducted in the Spanish region of Málaga (Andalucía) and approved by the North-East Malaga Ethics Committee (1078-N-20). Interviews were telephone-administered to guarantee the safest means to communicate during the COVID-19 pandemic. Researchers contacted participants by telephone, explained the study in detail, answered any questions that arose, and obtained consent from those willing to participate in the study [23].

### Ethics Approval and Consent to Participate

The study was approved by the North-East Malaga Ethics Committee (1078-N-20). Participants provided written consent before taking part.

### Trial Registration

This study was registered in ClinicalTrials.gov (NCT04385797).

### Setting

Participants were identified from the Support, Monitoring and Reminder Technology for Mild Dementia (SMART4MD; NCT03325699) [24] and TV-Based Assistive Integrated Service to Support European Adults Living with Dementia (TV-AssistDem; NCT03653234) [25] randomized clinical trials (RCTs), which aimed to assess the effects of ICTs to support MCI/MD using a tablet-based health application and a television-based assistive integrated service, respectively. In both RCTs, a broad definition of MCI, a subjective memory deterioration sustained over time, was considered. All participants had undergone 1-3 previous assessments (in 6-month intervals) in the RCTs on cognition, quality of life, and depression prior to the COVID-19 breakout.

### Participants

Researchers from the Biomedical Research Institute of Malaga contacted 210 potential respondents from the SMART4MD (n=111, 52.9%) and TV-AssistDem (n=99, 47.1%) RCTs by telephone. In total, 151 participants, SMART4MD (n=75, 49.7%) and TV-AssistDem (n=76, 50.3%), agreed to participate. However, for 8 (5.3%) of them, it was not possible to assess the main variable (cognition) and the secondaries variables (quality of life, perceived health status, and depression), because their abilities to answer the questionnaires were compromised during the time of assessment.

Participants were eligible for inclusion when the following criteria applied: participating in the SMART4MD and TV-AssistDem RCTs and agreeing to participate by giving consent. Eligibility criteria of the aforementioned RCTs were age>55 years or >60 years, perception of memory problems for at least 6 months, score of 20-28 or 23-27 points in the Mini-Mental State Exam (MMSE), independently living, having an informal caregiver, and taking care of their medical prescription. Patients with a score above 11 on the Geriatric Depression Scale (GDS), a terminal illness, or specific cognitive or physical conditions that would reduce their ability to use a tablet or a television were excluded.

### Interview Process

Participants were contacted by telephone by 5 health care professionals (2 neuropsychologists, 1 clinical psychologist, 1 psychologist, and 1 psychiatric and mental health clinical nurse specialist). Researchers had previously established relationships with participants during both RCTs. Quantitative and qualitative strategies were used to create an unstandardized ad hoc telephone-based survey in order to gather as much information as soon as possible. The exceptional situation did not allow us to test the instrument prior to its implementation by phone. To minimize the interference of this situation in the results, validated phone versions tests were used.

The survey was a useful tool for guiding the interviewers and gathering information simultaneously in a homogenous way. A model of the questionnaire used is attached in Annex 1 in Multimedia Appendix 1.

Researchers interviewed the participants between May 11 and June 26, 2020. The variables of sociodemographic data (age, sex, and living arrangements), health perception-management (change in living arrangements due to lockdown, presence of COVID-19 symptoms, frequency of access to COVID-19 information), sleep-rest patterns, types of ICTs (smartphone, tablet, television, laptop), and their uses (informative, cognitive, entertainment, and socialization) were collected from the participants unless their abilities to answer such a long interview were compromised, in which case the caregivers were interviewed on their behalf. The questionnaires that evaluated the main variables (cognition, quality of life, depression, perceived stress, and technophilia) were answered by the participants.

The mean time from the start of the lockdown and home confinement measures to the interview was 70.36 days (SD 12.40, range 52-102).

### Instruments Used Before and After the Lockdown

#### Cognition

The primary outcome variable was cognition. During the assessment prior to the COVID-19 outbreak (T0), the MMSE [26] was used to assess the cognitive function of the participants with MCI/MD. We decided to use as eligibility criteria a broad spectrum, because although the common cut-off score for cognitive impairment is 24, it has been shown that an MMSE cut-off score of 28 provides high sensitivity and specificity for detecting MD in a well-educated population with self-reported memory complaints [27].

During the COVID-19 outbreak (T1), the validated telephone version of the MMSE had to be used to maintain health and safety measures. This phone version has a maximum score of 22 because it cannot cover all sections [28]. For example, on the spatial orientation section, researchers were not able to check on which floor the patient was. Motor skills or some language skills could not be measured either. In the telephone version, the subject is asked only to repeat a phrase and name 1 item. The items of the original version, such as naming a second word, asking to follow a 3-stage command, reading and obeying a sentence, writing a sentence, or copying an intersecting pentagon, could not be measured.

Although the full version of the MMSE was used in the T0 assessment, for data analysis, the scoring was based on the 22 items of the phone version.

#### Quality of Life and Perceived Health Status

The health-related quality of life (HRQoL) of the participants was measured in both assessments using the total score of the Quality of Life-Alzheimer’s Disease Scale (QoL-AD) [29]. The QoL-AD is a 13-item measure, in which responses are 4-point multiple-choice options (1=poor, 2=fair, 3=good, 4=excellent). It includes questions related to the interpersonal, environmental, functional, physical, and psychological status of a person with dementia, and thus, it is a global measure for the quality of life. Scale scores range from 13 to 52, with higher scores indicating a greater quality of life. In cases where patients had compromised cognitive function, informal caregivers completed the QoL-AD in parallel and on behalf of the people with MCI/MD.

The European Quality of Life 5 Dimensions 3 Levels (EuroQoL-5D-3L) [30] was also administered in both assessments. Currently, the EuroQoL-5D-3L is 1 of the most widely used generic preference-based measures in the world. It assesses an individual’s HRQoL [31]. It has been shown to be valid in different patient groups and settings [32], including patients with cognitive impairment and dementia [33].

The EuroQoL-5D-3L consists of 5 questions along with a visual analog scale (VAS). The VAS records the patient’s self-rated health on a vertical scale, where the endpoints are “the best health you can imagine” and “the worst health you can imagine.” Due to the impossibility of the patients to see the VAS during the T1 assessment, they were asked to rate their health status. Only the VAS-perceived health status assessment was used for this study, combined with the Qol-AD.

#### Depression

The short form of the GDS was used during the T0 assessment [34]. It is a scale with 15 items and a range of scores, where 0-4 is considered normal, 5-8 indicates mild depression, 9-11 indicates moderate depression, and 12-15 indicates severe depression.

During the COVID-19 lockdown (T1), the telephone version of the GDS [35] was used. This version has high internal consistency and is highly correlated with the validated face-to-face administration of the scale, indicating that it is a valid instrument for screening depression among elderly people in special situations, such as the COVID-19 outbreak.

### Instruments Used Only After the Lockdown

#### Technophilia

To measure older people’s attitudes and enthusiasm toward technologies, the Instrument for Measuring Older People’s Attitudes Toward Technology (TechPH) was used during the T1 assessment [36]. This questionnaire is designed to specifically assess technophilia in the older population and includes 6 items assessed on a 5-point Likert scale from 1 (fully disagree) to 5 (fully agree). The scale has 2 factors to define technophilia: technology enthusiasm and technology anxiety. It refers to a person’s enthusiasm and positive feelings toward their technology use and the absence of fears and doubts about their ability to manage it.

#### Perceived Stress

The Perceived Stress Scale (PSS) [37] measures the degree to which situations in one’s life are appraised as stressful. The scale has 14 questions regarding feelings and thoughts during the past month and are rated according to frequency (0=never, 1=almost never, 2=sometimes, 3=fairly often, 4=very often). PSS scores are obtained by reversing responses (eg, 0=4, 1=3, 2=2, 3=1, 4=0) to the 7 positively stated items (items 4, 5, 6, 7, 9, 10, and 13) and then summing across the entire 14-scale item. A higher score indicates a higher level of perceived stress. This questionnaire was used only during the T1 assessment. Studies report that the reduced version, PSS-10, has optimal psychometric properties in the general population and people exposed to confinement to assess perceived stress [38,39].

#### Other Variables

Other variables were sociodemographic data, including age, sex, and living arrangements; health perception-management (ie, change in living arrangements due to lockdown, presence of COVID-19 symptoms, frequency of access to COVID-19 information); coping-stress tolerance (ie, self-perceived mental health and well-being and mood); sleep-rest patterns (ie, self-perceived alterations in usual sleep patterns); and types of ICTs (smartphone, tablet, television, laptop) and their uses (informative, cognitive, entertainment, and socialization).

The survey data followed Gordon’s Functional Health Patterns (Multimedia Appendix 1) [40]. To collect data on health perception, health management, and sleep-rest patterns, questions with numerically rated items were used. Open questions were included for the qualitative assessment of the patterns of coping-stress tolerance, activity-exercise, and role-relationship.

### Data Analysis

**Figure 1:**
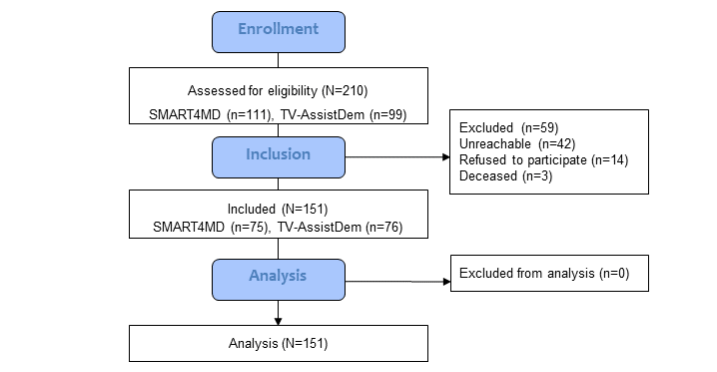
Participant flow diagram. SMART4MD: Support, Monitoring and Reminder Technology for Mild Dementia; TV-AssistDem: TV-based ASSistive Integrated Service to supporT European adults living with mild DEMentia or mild cognitive impairment.

The flow of participants is shown schematically with counts in a participant flow diagram (Figure 1). Statistics considered for presentation for continuous measures were the mean and SD, and if the criterion of normality was not met, the median and the first and third quartiles. Categorical variables were summarized using counts and percentages.

The change in means in the main outcome (cognition) and in the secondary outcomes (quality of life, perceived health status, and depression) were analyzed with respect to the last assessment of the RCTs (SMART4MD and TV-AssistDem) using the repeated measure *t* test or the nonparametric Wilcoxon test, if appropriate (considering significant values of α<.05). In the secondary outcomes, we applied Bonferroni correction for 3 comparisons (considering significant values of α<.017).

For the analysis of groups based on living arrangements (living alone vs living with others), the *t* test was used for continuous variables and the chi-square test for categorical variables. To evaluate the association of this variable with mental health outcomes, multivariate logistic regression for binary variables or linear regression for continuous variables was performed to adjust for confounders: age, sex, and technophilia. In addition, we explored the association between a change in living arrangements during the pandemic and cognition, depression, quality of life, and perceived health status with univariate and multivariate linear regression (adjusting for the following confounders: sex, age, and current living arrangements). For this comparison, we applied Bonferroni correction for 4 comparisons (considering significant values of α<.013).

For the analysis using groups based on the score in technophilia (based on the median of the TechPH index as the cut-off point), the *t* test or nonparametric Wilcoxon test was used for continuous variables and the chi-square test for categorical variables. To evaluate the association of this variable with mental health outcomes, multivariate logistic regression for binary variables or linear regression for continuous variables was performed to adjust for confounders: age, sex, and living arrangements.

To establish the Bonferroni correction for multiple comparisons (regarding living arrangements and technophilia groups), the number of independent tests was estimated with principal component analysis. Of the 37 variables analyzed, the first 35 components explained >99% (99.3%) of the variance. Thus, we considered the values significant with α<.0014.

To analyze the assumptions of all linear regression models, the Ramsey RESET linearity test, the Breusch-Pagan homoscedasticity test, and the Shapiro-Wilk normality test of the model residuals were used (see Annex 2 in Multimedia Appendix 1). When cognition, quality of life, health status, and depression were used as dependent variables, the assumption of the normality of the residuals of the model was not fulfilled, and transformation did not solve the problem, robust linear regression models were used (using the *robustbase* package and the *lmrob* function in R).

The R version 4.0.4 program was used for all statistical analysis [41].

## Results

### Participants

Of the 210 potential respondents (n=111 [52.9%] from SMART4MD and n=99 [47.1%] from TV-AssistDem), a total of 165 (78.6%) of 210 respondents was successfully reached, of which 151 (91.5%) agreed to participate (Figure 1). In addition, 150 (99.3%) participants completed the full interview without assistance, and 15 informal caregivers were interviewed on behalf of participants whose abilities to answer such a long interview were compromised. Of these 15 participants, 11 (73%) answered the main variables questionnaires and 4 (27%) were unable to do so. Given the complexity of MCI/MD, to not skew the results of patients with a greater decline, all data were considered.

The mean time between the last assessment of the RCTs (T0) and the interview during the lockdown (T1) was 199.33 days (SD 52.43, range 67-395), and the mean duration of the calls was 50.14 minutes (SD 16.40).

### Sociodemographics

The mean age of the sample was 74.31 years (SD 6.48), 97 (64.2%) of 151 participants were women, 36 (23.8%) lived alone, and 80 (53.3%) had high attraction to technology (high technophilia). The COVID-19 outbreak forced 22 (14.6%) of 151 participants to change their living arrangements (Table 1).

**Table 1:** Sample sociodemographic characteristics and differences between living alone and living with others, and high technophilia and low technophilia.

Characteristics		Total participants (N=151)	Living alone (n=36)	Living with others (n=115)	Statistics	*P* value	High technophilia (n=80)	Low technophilia (n=65)	Statistics	*P* value
Age (years), mean (SD)		74.31 (6.48)	76.31 (5.38)	73.69 (6.69)	*t*_93_=–2.14	.03	73.69 (6.33)	74.74 (6.63)	*t*_93_=0.97	.34
**Sex, n (%)**										
	Male	54 (35.8)	4 (11.1)	50 (43.5)	χ^2^_1_=12.50	<.001	31 (63.3)	18 (36.7)	χ^2^_1_=1.96	.16
	Female	97 (64.2)	32 (88.9)	65 (56.5)	χ^2^_1_=12.50	<.001	49 (51.0)	47 (49.0)	χ^2^_1_=1.96	.16
**Change in living arrangements, n (%)**										
	Yes	22 (14.6)	6 (16.7)	16 (13.9)	χ^2^_1_=0.17	.68	11 (13.8)	10 (15.4)	χ^2^_1_=0.08	.78
	No	129 (85.4)	30 (83.3)	99 (86.9)	χ^2^_1_=0.17	.68	69 (86.3)	55 (84.6)	χ^2^_1_=0.08	.78

### Differences in Cognition, Quality of Life, Perceived Health Status, and Depression Prior to and During the COVID-19 Outbreak

Regarding the differences between the period before and during the outbreak, there were no differences in the main outcome: cognition. After correction for multiple comparisons, there were no statistically significant differences in the quality of life, perceived health status, or depression between the 2 periods (Table 2).

**Table 2:** Differences in cognition, quality of life, perceived health status, and depression prior to and during the COVID-19 outbreak.

Outcomes			Before the COVID-19 outbreak	During the COVID-19 outbreak		Statistics		*P* value
**Main outcome**								
	Cognition (MMSE^a^), median (IQR)	19 (17-20)		19 (17-21)	Z=–0.798		.43	
**Secondary outcomes^b^**								
	QoL-AD^c^, mean (SD)	35.97 (4.74)		36.25 (5.44)	*t*_144_=–0.80		.43	
	Perceived health status (EuroQoL-5D-3L^d^ thermometer), median (IQR)	70 (50-80)		70 (60-85)	Z=–1.94		.05	
	Depression (GDS^e^), median (IQR)	3 (1-5)		2 (1-4)	Z=–0.01		.99	

^a^MMSE: Mini-Mental State Exam.

^b^Significant results with *P*<.02.

^c^QoL-AD: Quality of Life-Alzheimer's Disease Scale.

^d^EuroQoL-5D-3L: European Quality of Life 5 Dimensions 3 Levels.

^e^GDS: Geriatric Depression Scale.

### Differences Between Individuals Living Alone and Living With Others, and With Change in Living Arrangements

Regarding social isolation (living alone and living with others), after Bonferroni correction, there was no significant association between the variables of the study (Table 3). Some factors reached nominally significance: self-perceived fear (being alone 36.1% vs 18.4%; χ^2^=4.27; *P*=.04) and depression (being alone 3 vs 2; Z=–2.10; *P*=.04). In the multivariate models, depression did not reach nominally statistically significance (B=0.83; *P*=.06); see Table 3 (note: significant results with *P*<0.001). After Bonferroni correction, there were statistically significant differences regarding home visits, with those living alone (being alone 82.9% vs 51.3%; χ^2^=10.97; *P*<.001) receiving more visits than those living with others. There was no significant association regarding other variables (only a nominal significant association regarding the more frequent use of the newspaper in those living with others).

The change in living arrangements was not associated with cognition (unadjusted model: B=–0.21, *P*=.65; adjusted model: B=0.36, *P*=.44), quality of life (unadjusted model: B=–0.74, *P*=.40; adjusted model: B=1.05, *P*=.21), perceived health status (unadjusted model: B=3.34, *P*=.41; adjusted model: B=3.34, *P*=.41), or depression (unadjusted model: B=–0.15, *P*=.74; adjusted model: B=–0.25, *P*=.56). After correction for multiple comparisons, there was no association with less perceived stress (unadjusted model: B=–4.72, *P*=.01; adjusted model: B=–0.26, *P*=.02) but the results reached nominal significance. Annex 2 in Multimedia Appendix 1 shows more detailed information about linear regression models.

**Table 3:** Health perception-management, coping-stress tolerance, and sleep-rest functional health patterns during the COVID-19 outbreak and differences between living alone and with others.

Overall health status		Total participants (N=151)	Living alone (n=36)	Living with others (n=115)	Statistics	*P* value	Odds ratio (OR)/B^a^	*P* value
**Health status (COVID-19), n (%)**								
	No symptoms	147 (97.4)	35 (97.2)	112 (97.4)	χ^2^_4_=4.13	.13	—^b^	—
	Symptoms without test	3 (2.0)	0	3 (2.6)	χ^2^_4_=4.13	.13	—	—
	Symptoms and positive test	1 (0.7)	1 (2.8)	0	χ^2^_4_=4.13	.13	—	—
	Hospitalized	0	0	0	χ^2^_4_=4.13	.13	—	—
	Intensive care unit (ICU) inpatient	0	0	0	χ^2^_4_=4.13	.13	—	—
	Deceased	0	0	0	χ^2^_4_=4.13	.13	—	—
**Self-perceived mental health and well-being, n (%)**								
	Well	108 (71.5)	23 (63.9)	85 (73.9)	W_1_=1.27	.26	1.64	.27
	Calm	64 (42.7)	14 (38.9)	50 (43.9)	W_1_=1.20	.27	1.47	.37
	Sad	49 (32.7)	15 (41.7)	34 (29.8)	W_1_=0.05	.83	.91	.83
	Worried	69 (46.0)	20 (55.6)	49 (43.0)	W_1_=0.68	.41	.65	.31
	Afraid	34 (22.7)	13 (36.1)	21 (18.4)	W_1_=4.27	.04	.37	.04
	Anxious	33 (22.0)	9 (25.0)	24 (21.1)	W_1_=0.31	.58	.71	.49
	Bored	28 (18.7)	9 (25.0)	19 (16.7)	W_1_=1.84	.18	.46	.14
**Self-perceived sleep quality, n (%)**								
	Maintained	117 (77.5)	29 (80.6)	88 (76.5)	W_1_=0.12	.73	OR=1.07	.90
	Altered	34 (22.5)	7 (19.3)	27 (23.5)	W_1_=0.12	.73	OR=1.07	.90
Cognition (MMSE^c^), median (IQR)		19 (17-21)	19 (17-21)	19 (17-20.75)	Z=–0.57	.57	B^d^=0.30	.52
Quality of life (QoL-AD^e^, mean (SD)		36.25 (5.44)	35.03 (4.39)	36.66 (5.70)	*t*_143_=1.53	.13	B^d^=–1.88	.02
Perceived health status (EuroQoL-5D-3L^f^ thermometer), median (IQR)		70 (60-85)	75 (60-100)	70 (60-80)	Z=–1.51	.13	B^d^=6.67	.12
Depression (GDS^g^), median (IQR)		2(1-4)	3 (2-5)	2 (1-4)	Z=–2.10	.04	B^d^=0.83	.06
Perceived stress (PSS^h^), mean (SD)		19.5 (8.64)	20.44 (7.96)	19.19 (8.87)	*t*_149_=–0.75	.45	B^i^=0.08	.43

^a^Multivariate models (logistic or lineal) with living arrangements (living alone and living with others ) as the independent variable and gender, age, and technophilia (high technophilia and low technophilia) as covariates. More information about linear regression models is shown in Annex 2 in Multimedia Appendix 1.

^b^Not applicable.

^c^MMSE: Mini-Mental State Exam.

^d^Robust linear regression.

^e^QoL-AD: Quality of Life-Alzheimer's Disease Scale.

^f^EuroQoL-5D-3L: European Quality of Life 5 Dimensions 3 Levels.

^g^GDS: Geriatric Depression Scale.

^h^PSS: Perceived Stress Scale.

^i^As the residuals of the model were not normal, we transformed the dependent variable in its logarithmic form.

### Differences Between High- and Low-Technophilia Groups

After correction for multiple comparisons, there was no significant association between technophilia and the variables of the study. Only some variables reached nominal significant associations: self-perceived boredom (high technophilia 10.1% vs 27.7%; χ^2^=7.44; *P*=.01), calmness (high technophilia 31.6% vs 52.3%; χ^2^=6.30; *P*=.01), perceived stress (high technophilia 18.1 vs 21.23; *t*=2.19; *P*=.03), depression (high technophilia 2 vs 3; *Z*=2.16; *P*=.03), and quality of life (high technophilia 37.3 vs 35.3; *t*=2.24; *P*=.03). In the multivariate models, after controlling for possible confounders, the associations only maintained nominally statistical significance and the perceived health status reached nominal significance (B=6.44, *P*=.04); see Table 4 (note: significant results with *P*<.001).

**Table 4:** Health perception-management, coping-stress tolerance, and sleep-rest functional health patterns during the COVID-19 outbreak and differences between the high- and low-technophilia groups.

Overall health status		Total participants (N=151)	High technophilia (n=80)	Low technophilia (n=65)	Statistics	*P* value	Odds ratio (OR)/B^a^	*P* value
**Health status (COVID-19), n (%)**								
	No symptoms	147 (97.4)	78 (97.5)	63 (96.9)	χ^2^_2_=1.39	.50	—^b^	—
	Symptoms without test	3 (2.0)	2 (2.5)	1 (1.5)	χ^2^_2_=1.39	.50	—	—
	Symptoms and positive test	1 (0.7)	0	1 (1.5)	χ^2^_2_=1.39	.50	—	—
	Hospitalized	0	0	0	χ^2^_2_=1.39	.50	—	—
	Intensive care unit (ICU) inpatient	0	0	0	χ^2^_2_=1.39	.50	—	—
	Deceased	0	0	0	χ^2^_2_=1.39	.50	—	—
**Self-perceived mental health and well-being, n (%)**								
	Well	108 (71.5)	54 (76.9)	50 (67.5)	χ^2^_1_=1.57	.21	1.64	.20
	Calm	64 (42.7)	25 (31.6)	34 (52.3)	χ^2^_1_=6.30	.01	2.23	.02
	Sad	49 (32.7)	22 (27.8)	25 (38.5)	χ^2^_1_=1.83	.18	1.41	.36
	Worried	69 (46.0)	31 (39.2)	35 (53.8)	χ^2^_1_=3.06	.08	1.75	.11
	Afraid	34 (22.7)	17 (21.5)	16 (24.6)	χ^2^_1_=0.194	.66	1.21	.65
	Anxious	33 (22.0)	13 (16.5)	18 (27.7)	χ^2^_1_=2.67	.10	2.01	.10
	Bored	28 (18.7)	8 (10.1)	18 (27.7)	χ^2^_1_=7.44	.01	3.69	.01
**Self-perceived sleep quality, n (%)**								
	Maintained	117 (77.5)	66 (82.5)	47 (72.3)	χ^2^_1_=2.17	.14	1.92	.11
	Altered	34 (22.5)	14 (17.5)	18 (27.7)	χ^2^_1_=2.17	.14	1.92	.11
Cognition (MMSE^c^), median (IQR)		19 (17-21)	19 (17-21)	18 (16.25-21)	Z=–1.13	.26	B^d^=0.30	.52
Quality of life (QoL-AD^e^), mean (SD)		36.25 (5.44)	37.33 (5.48)	35.33 (4.90)	t_139_=2.24	.03	B^d^=1.64	.03
Perceived health status (EuroQoL-5D-3L^f^ thermometer), median (IQR)		70 (60-85)	80 (60-90)	70 (60-80)	*t*_142_=–1.65	.10	B^d^=6.44	.04
Depression (GDS^g^), median (IQR)		2(1-4)	2 (1-4)	3 (1-5)	Z=–2.16	.03	B^d^=–0.83	.03
Perceived stress (PSS^h^), mean (SD)		19.5 (8.64)	18.1 (8.77)	21.23 (8.21)	*t*_141_=2.19	.03	B^i^=–0.19	.02

^a^Multivariate models (logistic or lineal) with technophilia (high and low) as the independent variable and gender, age, and living arrangements (living alone and living with others) as covariates. More information about linear regression models is shown in Annex 2 in Multimedia Appendix 1.

^b^Not applicable.

^c^MMSE: Mini-Mental State Exam.

^d^Robust linear regression.

^e^QoL-AD: Quality of Life-Alzheimer's Disease Scale.

^f^EuroQoL-5D-3L: European Quality of Life 5 Dimensions 3 Levels.

^g^GDS: Geriatric Depression Scale.

^h^PSS: Perceived Stress Scale.

^i^As the residuals of the model were not normal, we transformed the dependent variable in its logarithmic form.

### Health Care and Social Support Services Access and Utilization and Informative-Related Uses of ICTs During the COVID-19 Outbreak

Of 148 participants, 39 (26.4%) reported accessing extreme and 32 (21.6%) reported accessing too much COVID-19 information. The most frequent ICT used to access COVID-19 information was mainly the television (134/147, 91.2%), and most participants were also informed through family and friends (120/148, 81.1%). Furthermore, only 46 (30.7%) of 150 participants did not contact health or social services (Table 5; note: significant results with *P*<.001).

**Table 5:** Health care and social support service access and utilization and informative-related uses of ICTs^a^ during the COVID-19 outbreak and differences between living alone and with others, and high and low technophilia.

Characteristic			Total participants (N=151)		Living alone (n=36)		Living with others (n=115)		Chi-square (*df*)		*P* value		High technophilia (n=80)		Low technophilia (n=65)		Chi-square (*df*)		*P* value
**COVID-19 information access, n (%)**																			
	None	3 (2.0)		1 (2.9)		2 (1.8)		2.42 (4)		.66		0		1 (1.6)		4.21 (4)		.38	
	Too little	33 (22.3)		8 (23.5)		25 (21.9)		2.42 (4)		.66		22 (27.5)		10 (15.9)		4.21 (4)		.38	
	Moderate	41 (27.7)		12 (35.3)		29 (25.4)		2.42 (4)		.66		20 (25.0)		19 (30.2)		4.21 (4)		.38	
	Too much	32 (21.6)		7 (20.6)		25 (21.9)		2.42 (4)		.66		16 (20.0)		16 (25.4)		4.21 (4)		.38	
	Extreme	39 (26.4)		6 (17.6)		33 (28.9)		2.42 (4)		.66		22 (27.5)		17 (27.0)		4.21 (4)		.38	
**COVID-19 information source, n (%)**																			
	Family and friends	120 (81.1)		29 (85.3)		91 (79.8)		0.51 (1)		.48		64 (80.0)		55 (87.3)		1.35 (1)		.25	
	Television	134 (91.2)		32 (94.1)		102 (90.3)		0.48 (1)		.49		70 (88.6)		61 (96.8)		3.31 (1)		.07	
	Smartphone	56 (38.1)		13 (37.1)		43 (38.4)		0.02 (1)		.89		34 (42.5)		22 (35.5)		0.72 (1)		.40	
	Tablet	12 (8.2)		1 (2.9)		11 (9.8)		1.73 (1)		.19		10 (12.7)		2 (3.2)		4.08 (1)		.04	
	Laptop	10 (6.8)		1 (2.9)		9 (8.0)		1.06 (1)		.30		6 (7.7)		4 (6.3)		0.10 (1)		.76	
	Newspaper	12 (8.2)		0		12 (10.6)		3.93 (1)		.05		7 (8.9)		5 (7.9)		0.04 (1)		.84	
	Digital media	71 (49.0)		16 (47.1)		55 (49.5)		0.07 (1)		.80		41 (52.6)		30 (48.4)		0.24 (1)		.62	
	Radio	37 (24.5)		10 (29.4)		27 (24.1)		0.39 (1)		.53		18 (22.8)		19 (30.6)		1.11 (1)		.29	
**Resources contacted, n (%)**																			
	None	46 (30.7)		10 (27.8)		36 (31.6)		0.19(1)		.67		21 (26.3)		21 (32.8)		0.74 (1)		.39	
	Health services	88 (58.3)		17 (47.2)		71 (61.7)		2.38 (1)		.12		46 (57.5)		38 (58.5)		0.01 (1)		.91	
	COVID-19 services	5 (3.3)		1 (2.8)		4 (3.5)		0.42 (1)		.84		4 (5.0)		1 (1.5)		1.29 (1)		.26	
	Emergency services	10 (6.6)		3 (8.3)		7 (6.1)		0.22 (1)		.64		5 (6.3)		5 (7.7)		0.12 (1)		.73	
	Social service nongovernment organization (NGO)	5 (3.3)		3 (8.3)		2 (1.8)		3.68 (1)		.06		4 (5.0)		1 (1.6)		1.25 (1)		.26	

^a^ICT: information and communications technology.

### Cognitive-, Entertainment-, and Socialization-related Uses of ICTs During the COVID-19 Outbreak

Although most of the participants (46/151, 30.7%) preferred paper-based memory exercises, the most frequent ICT used for cognition was the television (16/151, 10.7%). The most used ICTs for entertainment were the television (138/151, 92%), followed by the smartphone (60/151, 40%), and for socialization, the smartphone (75/151, 50.3%). Detailed information is given in Table 6 (note: significant results with *P*<.001).

**Table 6:** Cognitive-, entertainment-, and socialization-related uses of ICTs^a^ during the COVID-19 outbreak and differences between living alone and living with others, and high and low technophilia.

Activity category		Total participants (N=151)	Living alone (n=36)	Living with others (n=115)	Chi-square (*df*)	*P* value	High technophilia (n=80)	Low technophilia (n=65)	Chi-square (*df*)	*P* value
**Cognitive, n (%)**										
	Paper	46 (30.7)	14 (40.0)	32 (27.8)	7.52 (5)	.19	23 (28.7)	21 (32.8)	5.44 (5)	.36
	Smartphone	3 (2.0)	0	3 (2.6)	7.52 (5)	.19	3 (3.8)	0	5.44 (5)	.36
	Tablet	7 (4.7)	1 (2.9)	6 (5.2)	7.52 (5)	.19	3 (3.8)	4 (6.3)	5.44 (5)	.36
	Laptop	1 (0.7)	1 (2.9)	0	7.52 (5)	.19	0	1 (1.6)	5.44 (5)	.36
	Television	16 (10.7)	5 (14.3)	11 (9.6)	7.52 (5)	.19	11 (13.8)	5 (7.8)	5.44 (5)	.36
**Entertainment, n (%)**										
	Smartphone	60 (40.0)	13 (37.1)	47 (40.9)	0.16 (1)	.69	37 (46.3)	23 (35.9)	1.56 (1)	.21
	Tablet	18 (12.0)	1 (2.9)	17 (14.8)	3.61 (1)	.07	11 (13.8)	7 (10.9)	0.26 (1)	.61
	Laptop	20 (13.3)	3 (8.6)	17 (14.8)	0.90 (1)	.41	12 (15.0)	8 (12.5)	0.19 (1)	.67
	Television	138 (92.0)	31 (88.6)	107 (93.0)	0.73 (1)	.39	74 (92.5)	59 (92.2)	0.01 (1)	.94
**Socialization, n (%)**										
	Home visits	87 (58.8)	29 (82.9)	58 (51.3)	10.97 (1)	<.001	46 (57.5)	40 (63.5)	0.53 (1)	.47
	Smartphone	75 (50.3)	16 (45.7)	59 (51.8)	0.39 (1)	.53	47 (58.8)	27 (42.2)	3.90 (1)	.05
	Tablet	10 (6.8)	0	10 (8.8)	3.32 (1)	.12	7 (8.9)	3 (4.7)	0.95 (1)	.33
	Laptop	5 (3.4)	0	5 (4.4)	1.59 (1)	.59	3 (3.8)	2 (3.1)	0.04 (1)	.84
	Television	6 (4.0)	0	6 (5.3)	1.91 (1)	.34	5 (6.3)	1 (1.6)	1.96 (1)	.23

^a^ICT: information and communications technology.

## Discussion

### Principal Findings

This cohort study was conducted to understand the impact of restrictive measures in community-dwelling older adults with MCI and MD during the first COVID-19 outbreak.

Our findings show that the first months of the outbreak did not significantly impact the cognition, quality of life, perceived health status, and depression of our study population when making comparisons with baseline assessments prior to the outbreak. Change in living arrangements had no influence on these variables either. Living alone and technophilia were not associated with mental health–related variables after correction for multiple comparisons. However, being alone was nominally associated with self-perceived fear and depression, and higher technophilia with better quality of life, less boredom, perceived stress, and depression but also less calmness. Overall, health care and social support service access and utilization were high. The most used ICTs during the COVID-19 outbreak were the television for informative-, cognitive-, and entertainment-related uses and the smartphone for socialization.

### Comparison With Prior Work

To the best of our knowledge, few studies have addressed the consequences of the COVID-19 outbreak on the cognition of the elderly, and the use of technologies during this ongoing societal change.

Several studies have shown that quarantine measures have changed the behaviors and lifestyle of older people with cognitive decline [42], although in some cases, these changes have been less important than expected [43]. Lifestyle changes can increase the risk of dementia and cause cognitive impairment. However, our study showed that in our sample, the first stages of the COVID-19 outbreak did not cause significant cognitive decline in comparison with a previous assessment. This result could be explained by 2 reasons. On the one hand, the evaluation was carried out on an average of 70 days after the start of the home confinement restrictions and, likely, this time was not enough to influence cognitive decline. On the other hand, the data of our study show that most of the participants maintained an active lifestyle and used new technologies for cognitive stimulation, information access, leisure, and social connectedness. This combination of healthy lifestyle factors and opportunities for cognitive and social stimulation has proved to be important in reducing the risk of cognitive decline [44].

Regarding the lack of differences in the quality of life and perceived health status before and during the outbreak in people with MCI/MD, a similar conclusion was reached by another cohort study in a similar population in Spain using the EuroQoL-5D-3L [45]. Other studies on quality of life in different population groups during the pandemic have also not found a perception of poor quality of life in the elderly [46]. Our results could be explained because our sample has continued to carry out cognitive, entertainment, and social activities, which are associated with the definition of a good quality of life by the elderly [47]. Another factor that can lead to a good quality of life is maintaining well-being and good sleep quality. Poor sleep quality is known to have a significant impact on lower levels of life satisfaction and mood [48].

Regarding mental health, longitudinal studies have established that it is has been affected by the pandemic [49-51], but frequently, the impact has been higher in the younger population and those more economically vulnerable [51,52]. Likewise, another longitudinal study in Spain with a sample of older adults with dementia or cognitive impairment found an increase in depressive and anxious symptoms after the confinement [53]. Furthermore, attention has been drawn to the possible harmful effects of the excessively dramatic presentation of the consequences of the restrictions due to the pandemic, which are based mainly on survey studies, in many cases carried out without the required rigor [54]. However, the results are mixed, and, for example, some longitudinal studies in Spain [55] and Greece [56] did not find differences regarding depression when comparing the period before and after confinement, and another Dutch community study did not find an increase in depressive symptoms in the general population [57]. In a cross-sectional study comparing populations over and under the age of 60 years during the peak of the COVID-19 pandemic in Spain, the elderly did not demonstrate special vulnerability to acute stress and no sex differences were found. This study hypothesized a greater resilience in the elderly due to the economic and social difficulties experienced throughout their lives during the Spanish post–Civil War period (1939-1960), increasing their ability to cope with stress and face the pandemic resiliently [52]. The results of this study in which no differences were found in depressive symptoms could also be explained by the specific characteristics of the study sample, which present MCI and mainly maintain autonomy to live independently. In addition, the evaluations were conducted when the stricter measures of the first lockdown in Spain were being brought down. Moreover, the small incidence of the COVID-19 virus at the time of assessment could have influenced the results (infections less than 1%). These results show the complexity of the effects of the pandemic, highlighting the need for more longitudinal studies in different populations to evaluate the effects of the social restrictions and the pandemic.

Another factor to consider was whether living alone during COVID-19 confinement was associated with a higher prevalence of depression. Although several studies have found a significant association between depression and living alone during the pandemic [42,58], others have shown otherwise [59,60]. Our results are in line with the second ones, showing no significant differences in GDS scores. However, more studies are necessary to determine whether living alone is a risk factor for depression during the pandemic. The results are in line with our previous findings [61] that associated living alone with worse mental health at the beginning of the outbreak (without correcting for multiple comparisons). The way our sample used the ICTs, through online communication, remote social interactions, or video calls, could have been useful to address social isolation during the pandemic [62].

Regarding technophilia, our study did not find an association between a better attitude toward technology and better mental health. In line with these results, a multicenter study conducted in Norway, the United Kingdom, the United States, and Australia also found no change in loneliness and the quality of life in adults over 70 years who used ICTs to maintain social contact during the COVID outbreak [63]. However, we showed in a previous study based on the TV-AssistDem RCT how technology could be useful to maintain cognitive activities [61], and more studies need to clarify whether the evidence supports the recommendations on interventions that may improve the knowledge of ICTs and are related with the use of technology to maintain social connections and cognitive activities [64].

### Limitations

A main limitation of this study was changing the interview administration from face-to-face before the outbreak to telephonic during the outbreak. Interviews were performed by the same professionals in both cases to reduce this possible bias, the measures were rescaled accordingly, and the validated phone versions of the tests were used. In addition, the interview had a mean duration of 50.14 minutes, which could cause fatigue in this population and alter their performance.

Another limitation is that the sample came from 2 RCTs and the participants who agreed to participate in the RCTs may have special characteristics that make them not representative of the general population. Furthermore, the sample was from only 1 center in Andalusia. However, it is a larger sample than in other studies carried out in Spain to date with this type of population.

Moreover, 15 caregivers answered on behalf of patients whose ability to answer for themselves was compromised. Although they did not respond to the questionnaires that evaluated the main variables, their answers may have interfered with the results.

The Bonferroni correction for multiple comparisons is conservative and could have increased type II errors.

Some studies have pointed out that the effects of changes during the lockdown may be temporary compared to long-lasting ones. Therefore, future effects will need to be explored as it is possible that once the lockdown is over, many people may not return to their “normal routine” as before the pandemic and will continue to avoid face-to-face activities, especially those regarding social and physical activity due to the fear of the contagion [42].

### Conclusions

During the COVID-19 outbreak, governments’ restrictive measures demonstrated being effective in viral spread prevention. Although these restrictions have had negative effects on health and well-being and have changed lifestyles worldwide, our study showed how a presumably vulnerable population has shown more resilience to restrictive measures than expected. The people with MCI/MD did not experience a significant decline in cognition, quality of life, perceived health status, or depression during the period of the COVID-19 outbreak. The study also showed that being alone and a negative attitude toward technology are not associated with worse mental health after correcting for multiple comparisons. In addition, the data were collected over a short period, and further research is needed to explore whether maintaining restrictive measures for longer influences a worsening of cognitive abilities, quality of life, perceived health status, or depression and which factors increase the risk of poor mental health in this population. They reported overall well-being, maintained sleep quality, and presented moderate perceived stress. This could be related to the fact that our sample continued participating in daily activities, which plays a crucial role in enhancing and maintaining cognition [65,66], just like keeping social interaction. The use of ICTs was essential to carry out these activities during the restrictions. Television was the most widely used ICT for informational-, cognitive-, and entertainment-related uses, and the smartphone for socialization.

## Appendix

Multimedia Appendix 1 Model of the questionnaire used.

## Abbreviations

EuroQoL-5D-3L: European Quality of Life 5 Dimensions 3 Levels
GDS: Geriatric Depression Scale
HRQoL: health-related quality of life
ICT: information and communications technology
MCI: mild cognitive impairment
MD: mild dementia
MMSE: Mini-Mental State Examination
PSS: Perceived Stress Scale
QoL-AD: Quality of Life-Alzheimer’s Disease Scale
RCT: randomized clinical trial
SMART4MD: Support, Monitoring and Reminder Technology for Mild Dementia
TechPH: Instrument for Measuring Older People’s Attitudes Toward Technology
TV-AssistDem: TV-Based Assistive Integrated Service to Support European Adults Living with Dementia
VAS: visual analog scale
