# Supplementary material for: The Impact of COVID-19 Confinement on Cognition and Mental Health and Technology Use Among Socially Vulnerable Older People: Retrospective Cohort Study
Source: J Med Internet Res. 2022 Feb 22;24(2):e30598. doi: 10.2196/30598 (PMC8865547; doi:10.2196/30598)
Supplement: Multimedia Appendix 1 [file jmir_v24i2e30598_app1.docx]

**Annex 1. Survey questions regarding the experience of coronavirus disease confinement in community-dwelling older adults with mild cognitive impairment or mild dementia**

**INFORMED CONSENT**

Dear Mr./Mrs. “name of PMCI/MD”, I am “research name” of the “TV-AssistDeM/ SMART4MD” study in which you were participating. We would like to carry out another research, to study the effects of home confinement on your health and well-being. Participation is completely voluntary, and we will comply with the same ethical-legal considerations as the current study. During a half-hour interview we will ask you questions regarding your experience during confinement due to COVID-19. By agreeing to participate, you will help us better understand how community-dwelling older adults with memory problems handle home confinement. Understanding this will help services develop effective preventive programs and treatments that help people manage similar situations. It may be unpleasant to report these experiences, if you think that completing this interview may affect you in any way, we encourage you not to participate. Thank you for your cooperation.

**1. RESPONDENT**

1. Person with mild cognitive impairment or mild dementia
2. Caregivers on behalf of person with mild cognitive impairment or mild dementia whose cognitive or emotional status is compromised

**2. GENERAL INFORMATION**

2.1. Have your living arrangements changed due to confinement?

1. Yes
2. No

2.2. Regarding your living arrangements, who are you currently living with?

1. Alone
2. Spouse
3. Children
4. Spouse and children
5. Other

2.3. Regarding your partners, what is his/her working situation?

1. Previous unemployment
2. Presential work
3. Remote work
4. Work interruption
5. Job layoff
6. Retired
7. Student
8. Other

2.4. Regarding your partnert’s health status and COVID-19 which of the following applies:

1. He/She has/has had no symptoms
2. He/She has/has had symptoms compatible with COVID-19 but have not had a test done
3. He/She has/has had symptoms compatible with COVID-19 and have had a test done
4. He/She is/has been hospitalized because of COVID-19
5. He/She is/has been in an ICU because of COVID-19

2.5. Dou you have at-risk partner living with you? Elderly, chronically ill, etc?

1. No
2. Yes, (How many?)

**3. HEALTH PERCEPTION-HEALTH MANAGEMENT PATTERN**

## 3.1 Regarding your health status and COVID-19 which of the following applies:

1. I have/have had no symptoms
2. I have/have had symptoms compatible with COVID-19 but have not had a test done
3. I have/have had symptoms compatible with COVID-19 and have had a test done
4. I am/have been hospitalized because of COVID-19
5. I am/have been in an ICU because of COVID-19
6. Patient’s death

## 3.2. Regarding your health management, who is doing the groceries during confinement?

1. I am
2. A family member
3. A home worker
4. We are doing online shopping
5. Other

3. 3. How would you consider your access to COVID-19 information?

1. None
2. Too little
3. Moderate
4. Too much
5. Extreme

3.4. Which information source are you using to access COVID-19 information?

1. Family and friends
2. TV
3. Newspaper
4. Digital media
5. Radio

3.5. How would you consider your understanding of the COVID-19 information accessed?

1. None
2. Too little
3. Moderate
4. Too much
5. Extreme

3.6. Have you contacted any healthcare services to manage your health status, demand, change and/or cancel medical appointments, renew medical prescriptions, etc.?

1. Yes
2. No

3.7. Do you need contact information regarding healthcare services to manage your health status, demand, change and/or cancel medical appointments, renew medical prescriptions, etc.?

1. Yes
2. No

3.8. Have you contacted COVID-19 services to receive council regarding changes in your health status related to COVID-19?

1. Yes
2. No

3.9. Do you need contact information regarding COVID-19 services to receive council regarding changes in your health status related to COVID-19?

1. Yes
2. No

3.10. Have you contacted emergency services to receive assistance regarding changes in your health status related to COVID-19?

1. Yes
2. No

3.11. Do you need information contact information regarding emergency services to receive assistance regarding changes in your health status related to COVID-19?

1. Yes
2. No

3.12. Have you contacted any social support services to request support regarding food or medication provision?

1. Yes
2. No

3.13. Do you need contact information regarding social support services to request support regarding food or medication provision?

1. Yes
2. No

3.14. Do you receive any (formal) family support?

1. No
2. Yes (Previous and maintained)
3. Yes (Previous and cancelled)
4. Yes (New)

3.15. How often is the (informal) family support?

1. No
2. Daily
3. Weekly
4. Bi-weekly
5. Monday-Friday
6. Weekend
7. Monthly
8. Other

3.16. Do you receive support from any social services? (city council, social department, etc?

1. No
2. Yes (Previous and maintained)
3. Yes (Previous and cancelled)
4. Yes (New)

3.17. How often is the (informal) family support?

1. No
2. Daily
3. Weekly
4. Bi-weekly
5. Monday-Friday
6. Weekend
7. Monthly
8. Other

**4. COPING-STRESS TOLERANCE PATTERN**

4.1. Regarding your mental health and well-being during confinement, how would you say you are feeling?

CATEGORIES: Well, Calm, Sad, Worried, Afraid, Anxious, Bored.

**5. SLEEP-REST PATTERN**

5.1. How would is your sleep during confinement when comparing it to before?

1. Maintained
2. Altered
3. Does not know/answer

**6. ACTIVITY-EXERCISE PATTERN**

6.1. Which physical activities are you doing during confinement?

CATEGORIES: None, Walks, Stair climbing, Gymnastics, House chores.

6.2. Which intellectual activities are you doing during confinement?

CATEGORIES: Memory exercises, Reading, Playing games, Needlework, Painting

6.3. Which recreational activities are you doing during confinement?

CATEGORIES: Watching TV, Listening to radio or music, Playing with ICTs, House chores, Keeping pets or plants

**7. ROLE-RELATIONSHIP PATTERN**

7.1. How do you relate to loved ones? (open-ended question) (categorize several possible options)

7.2. Have you stopped receiving visitors?

1. No
2. Yes

7.3 (If yes) From whom have you stopped receiving visits?

1. Children
2. Grandchildren
3. Children and grandchildren
4. Siblings
5. Friends
6. Other

7.4. Are you contacting by phone?

1. No
2. Yes

7.5. Are you contacting by video call?

1. No
2. Yes

7.6. Are you contacting by phone?

1. No
2. Yes

7.7. Are you contacting by WhatsApp?

1. No
2. Yes

7.8. What technological devices do you use to contact loved ones?

CATEGORIES: (smartphone, tablet, TV, laptop/computer)

Annex 2

Linear Regression Model: dependent variable: log(PSS). Independent variable technophilia and living alone. Covariates: Age and Sex.

Breusch-Pagan test; BP = 1.2997, df = 1, p-value = 0.2543

RESET test; RESET = 0.18318, df1 = 8, df2 = 130, p-value = 0.9929

Shapiro-Wilk normality test of the residuals; W = 0.9897, p-value = 0.3754

Linear Regression Model: dependent variable: log(PSS). Independent variable change in living arrangement. Covariates: Age, Sex and current living arrangement.

Breusch-Pagan test; BP = 0.75387, df = 1, p-value = 0.3853

RESET test; RESET = 0.30775, df1 = 8, df2 = 130, p-value = 0.962

Shapiro-Wilk normality test of the residuals; W = 0.98178, p-value = 0.05412
